# DDX3X RNA helicase affects breast cancer cell cycle progression by regulating expression of KLF4

**DOI:** 10.1002/1873-3468.13106

**Published:** 2018-06-21

**Authors:** Ester Cannizzaro, Andrew John Bannister, Namshik Han, Andrej Alendar, Tony Kouzarides

**Affiliations:** ^1^ Department of Pathology and Gurdon Institute University of Cambridge Cambridge UK

**Keywords:** breast cancer, cell cycle, DDX3X, KLF4, RNA helicase

## Abstract

DDX3X is a multifunctional RNA helicase with documented roles in different cancer types. Here, we demonstrate that DDX3X plays an oncogenic role in breast cancer cells by modulating the cell cycle. Depletion of DDX3X in MCF7 cells slows cell proliferation by inducing a G1 phase arrest. Notably, DDX3X inhibits expression of Kruppel‐like factor 4 (KLF4), a transcription factor and cell cycle repressor. Moreover, DDX3X directly interacts with *KLF4* mRNA and regulates its splicing. We show that DDX3X‐mediated repression of KLF4 promotes expression of S‐phase inducing genes in MCF7 breast cancer cells. These findings provide evidence for a novel function of DDX3X in regulating expression and downstream functions of KLF4, a master negative regulator of the cell cycle.

## Abbreviations

**ChIP**, chromatin immunoprecipitation

**CLIP**, cross‐linking immunoprecipitation

**KLF4**, Kruppel‐like factor 4

**PI**, propidium iodide

DEAD‐BOX (DDX) RNA helicases belong to a large family of proteins characterized by the presence of a DEAD/H (Asp‐Glu‐Ala‐Asp/His) motif. Their main function is to unwind double stranded RNA to promote downstream molecular events. Despite a very high structural conservation of their functional helicase core, individual members of this protein family perform very different functions in RNA metabolism, all of which require multistep association/dissociation of large ribonucleoprotein complexes and modulation of RNA structures 1, 2, 3.

DEAD‐box proteins function as transcriptional regulators 4 and interact with spliceosome components 5, 6. They also mediate ribosome biogenesis 7, RNA export 8, translation initiation and termination 9, 10, 11 and mRNA degradation 12. Depending on the cellular and signalling context, the outcome of DDXs’ activity on their target RNAs can either promote gene expression or repress it.

Given the widespread activity of DEAD‐box helicases in regulating gene expression at different levels, and the numerous studies reporting their involvement in cellular growth 1, it is not surprising that alterations in their expression or function can potentially affect normal cellular homeostasis and contribute to cancer development and progression 13.

Ded1/DDX3 is one of the most widely studied and evolutionary conserved DEAD‐box RNA helicase subfamilies 14, 15. The human genome encodes two DDX3 homologues located on the sex chromosomes: DDX3X and DDX3Y 16, 17, 18. DDX3X is ubiquitously expressed in all tissues 15, 18 (https://www.proteinatlas.org/ENSG00000215301-DDX3X/tissue) and functional studies have implicated this protein in multiple processes of RNA regulation. Although DDX3X is predominantly localized within the cytoplasm, which is consistent with a translational regulation function 14, evidence supporting nuclear functions of the helicase (including roles in transcription, pre‐mRNA splicing, and mRNA export) also exists 14, 16.

The role of DDX3X in cancer biology has been studied in numerous different cancer models and altered expression of DDX3X or mutations in the *DDX3X* locus, have been correlated to cancer in different tissues 19, 20, 21, 22, 23, 24. In *in vitro* studies, the oncogenic activity of DDX3X has been mainly related to the control of cell growth, cell cycle progression and cell motility, but the mechanisms and the pathways through which DDX3X regulates these biological processes have only been partially uncovered.

The role of DDX3X in breast cancer has been suggested by both *in vitro* and clinical studies. *In vitro*, over‐expression of DDX3X in nontumourigenic breast epithelial cells (MCF10A) results in increased invasive cell properties 25. Conversely, knockdown of DDX3X decreases cell proliferation and clonogenic cell capacity in breast cancer cell lines (MCF‐7 and MDA‐MB‐231) 26, 27. Importantly, studies in patients’ samples highlighted the oncogenic potential of DDX3X overexpression by showing it correlates with distant breast cancer metastases formation and a worse overall patient survival 28. However, although these findings suggest a role for DDX3X in breast cancer, detailed investigations into its biological and molecular functions have not been performed.

Here, we report a mechanism through which DDX3X regulates the cell cycle of breast cancer MCF7 cells. We find that DDX3X functions to repress expression of Kruppel‐like factor 4 (KLF4), a zinc finger‐containing transcription factor and cell cycle repressor associated with growth arrest. Knockdown of DDX3X in MCF7 breast cancer cells induces expression of KLF4, alters *KLF4* mRNA exon usage and down‐regulates cell cycle factors genes, *CCNA2* and *CDK2*, leading to reduced cell growth. Thus, DDX3X promotes MCF7 cell proliferation, at least in part, by inhibiting expression of the key negative cell cycle regulator, KLF4. These findings are consistent with DDX3X playing an oncogenic role in MCF7 cells.

## Materials and methods

### Cell lines and mammalian tissue culture

Human transformed breast epithelial cancer cell line MCF7 and human breast epithelial metastatic cell line MDA‐MB‐231 were cultured in Dulbecco's modified eagle medium (DMEM) (Invitrogen, Carlsbad, CA, USA) both supplemented with 10% (v/v) FBS and 1% (w/v) penicillin/streptomycin/glutamine. Human immortalized nontumourigenic breast epithelial MCF10A cells were cultured in DMEM/F12 (Invitrogen) supplemented with 5% (v/v) horse serum, 20 ng·mL^−1^ EGF, 0.5 mg·mL^−1^ hydrocortisone, 100 ng·mL^−1^ cholera toxin, 10 μg·mL^−1^ insulin and 1% (w/v) penicillin/streptomycin.

### Cell growth assays

For cell proliferation assay, cells were seeded at 3 × 10^4^ in 2 mL of complete medium in three biological replicates, 24 h after transfection with relevant siRNAs. Cells were counted at days 2, 4 and 6 after plating using a Countess II cell counter. Proliferation curves and standard deviations were generated using the prism 7 (GraphPad Software, La Jolla, CA, USA) statistical tool.

For clonogenic assay, cells were seeded at 5 × 10^3^ in 2 mL of complete medium in three biological replicates, 24 h after transfection with relevant siRNAs. Cells were stained in crystal violet staining solution [0.5% (w/v) crystal violet powder, 20% (v/v) ethanol] after 6 days and plates were imaged using a ChemiDoc (BIORAD).

### Transient knockdown by RNA interference

MCF7, MCF10A and MDA‐MB‐231 cells were plated in a 6‐well tissue culture dish in the relevant culture media without antibiotics for 24 h reaching 60–80% confluency. Cells were then transfected with 100 nM final concentration siRNA using Dharmafect I reagent following the manufacturer's (Dharmacon, Lafayette, CO, USA) instructions. The following ON‐TARGET plus human siRNAs from Dharmacon were used: hDDX3X siRNA#6: J‐006874‐06; hDDX3X siRNA#8: J‐006874‐08; hKLF4 siRNA: J‐005089‐08; Nontargeting siRNA (scrambled sequence): D‐001810‐01. Cells were harvested 72 h after transfection and RNA and/or proteins were extracted for downstream assays.

### Generation of stable knockdown cell lines through shRNA lentiviral transduction

Stable DDX3X knockdown MCF7 cells were obtained by transduction of lentiviral particles bearing pLKO.1 plasmids expressing the relevant shRNA and the puromycin selection cassette (hDDX3X shRNA: TRCN0000000001; pLKO.1‐puro NonMammalian shRNA Control Plasmid DNA: SHC002).

MCF7 cells were plated in normal DMEM in 10 cm plate to reach 70% confluency the next day. The viral suspension (~6 mL) was supplemented with 8 μg·mL^−1^ human polybrene (Millipore, Burlington, MA, USA) and added to MCF7 target cells. Cells were left at 37 °C, 5% (v/v) CO_2_ overnight. Cells were selected in puromycin‐containing media and used for CLIP–qPCR assays 72 h after selection.

### Transient overexpression of human *KLF4*

MCF7 cells were plated in a 6‐well tissue culture dish in the relevant culture media without antibiotics for 24 h reaching 60–80% confluency. FuGENE transfection reagents were used following manufacturer's instructions (Promega, Madison, WI, USA). Increasing amounts (0 μg, 0.5 μg, 1 μg, 2 μg) of a pPB‐CAG‐hKLF4‐pA‐pgk‐Hygro plasmid, combined with decreasing amounts of pGG131 control plasmid (to hold the total amount of DNA at 2 μg in all conditions) were transfected. Cells were harvested 48 h after transfection for RNA extraction or processed for cells cycle analysis.

### Cell cycle assessment

MCF7 cells transfected with either scrambled siRNA or DDX3X targeting siRNA (#6 or #8). After 72 h they were then treated with 50 ng·mL^−1^ nocodazole for a further 16 h (overnight). Cells were detached by trypsinization, washed in 1X PBS containing 10 mm EDTA and pelleted by centrifugation at 120 ***g*** for 2 min. Pellets were resuspended in 100 μL 1X PBS containing 10 mm EDTA and 1% (w/v) BSA. 200 μL of propidium iodide (PI) staining solution [1% (v/v) NP‐40, PI 20 μg·mL^−1^, RNaseA 0.1 mg·mL^−1^ in 1X PBS, 10 mm EDTA, 1% (w/v) BSA] were added to resuspended pellets. Samples were kept on ice and measured using a FACSCalibur Cytometer (BD). At least 25 000 events per sample were collected. Data were processed with flowjo software. Standard deviation (error bars) and *P*‐values were calculated using the prism 7 statistical tool.

### Quantitative real‐time PCR for gene expression quantification

Total RNA was purified from cells using a miRNeasy Mini Kit (Qiagen, Hilden, Germany) following the manufacturer's instructions. Excess contaminating DNA was removed by on‐column DNase digestion using the RNase‐Free DNase Set (Qiagen), following the manufacturer's instructions.

First strand cDNA synthesis was performed using Superscript III Reverse Transcriptase kit (Invitrogen) according to the manufacturer's instructions.

Quantitative real‐time PCR was performed using an ABI StepOnePlus (Applied Biosystems, Foster City, CA, USA) sequence detection system using SYBR^®^green. PCR amplification was performed with an initial step of 10 min at 95 °C, followed by 40 cycles of 15 s at 95 °C and 1 min at 60 °C. Relative quantification was obtained calculating ∆∆Ct. *GAPDH* expression was used as reference gene. Standard deviation (error bars) and *P*‐values were calculated using the prism 7 statistical tool.

The following primers were used:

**Table:** 

*DDX3X*	fw: ACTATGCCTCCAAAGGGTGTCC	rev: AGAGCCAACTCTTCCTACAGCC
*CCNA2*	fw: CATGGACCTTCACCAGACCT	rev: GATTTAGTGTCTCTGGTGGGTTG
*CDK2*	fw: CATTCCTCTTCCCCTCATCA	rev: GCCCCCTCTGTGTTAATAAGC
*KLF4*	fw: CCATCCTTCCTGCCCGATC	rev: CGTCTTCCCCTCTTTGGCTT
*CCNE1*	fw: CAGATGAAGAAATGGCCAAAA	rev: TTTGGGTAAACCCGGTCAT
*GAPDH*	fw: TGCACCACCAACTGCTTAGC	rev: GGCATGGACTGTGGTCATGAG

### Chromatin immunoprecipitation and analysis of the *KLF4* gene

Two independent biological replicates were produced for each condition. Briefly, 72 h after siRNA transfection, MCF7 cells were cross‐linked with 1% (v/v) formaldehyde for 15 min. at room temperature and cross‐linking was stopped by the addition of 0.125 m glycine. Cells were then lysed in 1% (w/v) SDS, 10 mm EDTA, 50 mm Tris‐HCl pH 8.0, 1 mm sodium orthovanadate and protease inhibitors. Cells were sonicated in a Bioruptor Pico (Diagenode, Seraing, Belgium) to achieve a mean DNA fragment size of 500 bp. Immunoprecipitation was performed with relevant antibodies [5 μg anti‐RNA polymerase II antibody, clone CTD4H8 (Millipore, 05‐623) and control 5 μg GFP‐ChIP Grade (Abcam, Cambridge, UK, ab290)] for a minimum of 12 h at 4 °C in modified RIPA buffer [1% (v/v) Triton X‐100, 0.1% (w/v) deoxycholate, 0.1% (w/v) SDS, 90 mm NaCl, 10 mm Tris‐HCl pH 8.0, 1 mm sodium orthovanadate and EDTA‐free protease inhibitors]. An equal volume of protein A and G Dynabeads were used to bind the antibody and associated chromatin for 2 h at 4 °C. The beads were extensively washed prior to elution of the antibody bound chromatin. Reverse cross‐linking of DNA was followed by RNAse and Proteinase‐K treatment and DNA was purified using the Chip DNA Clean and Concentrate kit (Zymo Research, Irvine, CA, USA). Immuno‐precipitated DNA was analysed on an ABI StepOnePlus real‐time PCR instrument, using power SYBR^®^green PCR Mastermix according to the manufacturer's instructions. The chromatin immunoprecipitation efficiency was calculated as percentage of input normalized to the internal control for RNAPII occupancy, represented by *GAPDH* house‐keeping gene promoter region. Standard deviation (error bars) was calculated using the prism 7 statistical tool.

The following primers were used for ChIP analysis of *KLF4 (A‐E)* and *GAPDH*:

**Table:** 

*Amplicon A*	fw: TTACCCAGTGGACTTGC	rev: AACCAATGACCCTCTCAACTA
*Amplicon B*	fw: GGAAAGGGCTTCGAGATG	rev: AGCACTGTGCAGCGTGAACTG
*Amplicon C*	fw: GAGCGACGAGAGCGGACTCCT	rev: CCGTACTCACCGCCATTGTC
*Amplicon D*	fw: TCCCATCTTTCTCCACGTTC	rev: CGGGGACTGGTGAAGACC
*Amplicon E*	fw: CGGCTCTGTAACACCATAA	rev: TAGCACCCAGCCTACATTAAC
*GAPDH*	fw: GCGCACGTAGCTCAGGCC	rev: GAGCAGAGAGCGAAGCGGG

### RNA‐seq libraries preparation

Total RNA was extracted (as described above) from MCF7 cells transfected with either a scrambled siRNA or DDX3X targeting siRNA (#6 or #8) and harvested 72 h after transfection. Three independent biological replicates were produced for each condition.

Ribosomal RNA was depleted using a Ribo‐Zero rRNA removal kit (Human/Mouse/Rat) from Illumina(R), following the manufacturer's instructions. RNA‐seq libraries were produced using NEXTflex RNA‐Seq kit from Bio Scientific, following the manufacturer's instructions. Before multiplexing, excess primer was removed with AMPure XP beads (Beckman Coulter, Brea, CA, USA). Before and after multiplexing, libraries were tested for both size and quantity of DNA using a Qubit dsDNA HS assay kit and a high sensitivity D1000 ScreenTape system following the manufacturer's instructions.

### Bioinformatic analysis

For differential gene expression analysis in DDX3X knockdown MCF7 cells, trimmed reads were mapped in paired end mode to the h38 human genome using tophat 29 with the following parameters (–no‐coverage‐ search –max‐multihits 300 –report‐secondary‐alignments –read‐ mismatches 2 –library‐type fr‐firststrand). Multihits (reads mapping to multi loci) were filtered, along with reads mapping with quality score less than 20. Reads were counted across gene models taken from the Ensembl v86 gtf gene model list using the summarizeOverlaps function from the GenomicAlignments package in r 30. The strand of each read was inverted prior to counting to account for the fact that libraries represent the first strand of synthesised cDNA. Read counts were converted into normalized fragments per kilobase mapped (FPKM) values for quality control plots. Differential expression analysis was conducted on the raw count data using the DESeq2 package in r 31. *P* values were corrected for multiple testing using the Benjamini and Hochberg FDR correction. Significantly changing genes were identified based on a fold change greater than twofold (up or down) and an adjusted *P* value less than 0.05. In addition, significant genes were filtered to remove genes where both the control and mutant samples had an average FPKM score less than 1.

Gene Ontology analysis was performed by using default settings of DAVID tool 32.

Bioconductor DEXseq tool using default parameters 33 was used to identify differential exon usage between conditions, indicating differences in gene splicing between the conditions. This analysis indicates differences in gene splicing between conditions by including exons as terms in the model and looking for genes whereby differences between the exons accounts for a significant proportion of the variation between the conditions.

### Protein purification, detection and analysis

Cells were lysed by the addition of 1X SDS loading buffer [200 mm Tris‐HCl pH6.8, 20% (v/v) β‐mercaptoethanol, 2% (w/v) SDS, 0.1% (w/v) bromophenol blue, 40% (w/v) glycerol]. The lysates were sonicated using a VibraCell probe sonicator (Sonics) for 20 s at 22% amplitude. The samples were denatured by boiling for 5 min. and analysed by SDS/PAGE and western blotting. The following antibodies were used in the indicated concentrations for western blot: anti‐DDX3 mouse monoclonal [Abcam ab196032 (1 : 1000)]; anti‐β‐tubulin rabbit polyclonal [Abcam ab6046 (1 : 1000)]; anti‐GAPDH rabbit polyclonal [Abcam ab9483 (1 : 1000)]; anti‐KLF4 rabbit polyclonal [Abcam ab106629 (1 : 1000)].

### CLIP (UV Cross‐linking immunoprecipitation)‐qPCR

Cross‐linking immunoprecipitation‐qPCR was performed by adapting a published protocol for transcriptome‐wide iCLIP 34. MCF7 cells were cultured in 10‐cm diameter dishes. When cells reached confluence, media was removed, and cells were washed twice with ice‐cold 1X PBS. Ice ‐old 1X PBS was added, plates were placed on ice and irradiated once at 150 mJ·cm^−2^ at 254 nm. Cells were scraped and harvested into Eppendorf tubes and centrifuged at 15 800 ***g*** for 10 s at 4 °C. Cell pellets were resuspended in CLIP lysis buffer [50 mm Tris‐HCl, pH 7.4, 100 mm NaCl, 1% (v/v) Igepal CA‐630, 0.1% (w/v) SDS, 0.5% (w/v) sodium deoxycholate] supplemented with protease inhibitor cocktail (Complete™, Roche, Basel, Switzerland) and sonicated once using a VibraCell probe sonicator (Sonics, Newtown, CT, USA) for 20 s at 22% amplitude. Lysates were treated with 2 μL of Turbo DNase (Life Technologies, Carlsbad, CA, USA) for 3 min. at 37 °C with shaking at 1,100 rpm and then immediately placed on ice. Samples were then centrifuged for 10 min at 22 000 ***g*** at 4 °C to clear the lysate. The supernatant was collected and quantified using a bicinchoninic acid **assay** (BCA). Protein G Dynabeads were previously washed twice in lysis buffer and then resuspended in 100 μL lysis buffer with the relevant antibody (3 μg anti‐DDX3 mouse monoclonal‐Abcam‐ab196032; 3 μg mouse IgG2a Isotype Control Abcam‐ab18413) and incubated in rotation at room temperature for 1 h for antibody absorption. The lysates were added to the antibody/beads mix and incubated overnight with rotation at 4 °C. The next day beads were washed four times in high salt buffer [50 mm Tris‐HCl, pH 7.4, 1 m NaCl, 1 mm EDTA 1% (v/v) Igepal CA‐630, 0.1% (w/v) SDS, 0.5% (w/v) sodium deoxycholate] and twice in PK buffer (100 mm Tris‐ HCl pH 7.4; 50 mm NaCl; 10 mm EDTA). To digest the protein covalently bound to the RNAs and release peptide‐–RNA complexes, beads were resuspended in 50 μL PK buffer and 5 μL proteinase K (Roche, 03115828001) and incubated in shaking at 1,100 rpm for 30 min at 37 °C. RNA was extracted from the peptide–RNA complexes using a miRNeasy Mini Kit (Qiagen) following the manufacturer's instructions. Excess contaminating DNA was removed by on‐column DNase digestion using the RNase‐Free DNase Set (Qiagen), following the manufacturer's instructions. First strand cDNA synthesis was performed using Superscript III Reverse Transcriptase kit (Invitrogen) according to the manufacturer's instructions.

The following primers were used for detection of *PUS1* mRNA negative control: fw: TGGTGAGGACATGAGGAAAATG; rev: GAATGTGAGAGGGAAGGTGG.

RNA enrichment was quantified as percentage of Input. Standard deviations (error bars) and *P*‐values were calculated using the prism 7 statistical tool.

## Results

### DDX3X is required for proliferation of breast cancer cells

In order to gain insight into the biological role of DDX3X and investigate the specific requirement of DDX3X function in breast cancer cells, we first analysed the proliferation rates of MCF7 breast epithelial cancer cells upon knockdown of DDX3X.

**Figure 1:**
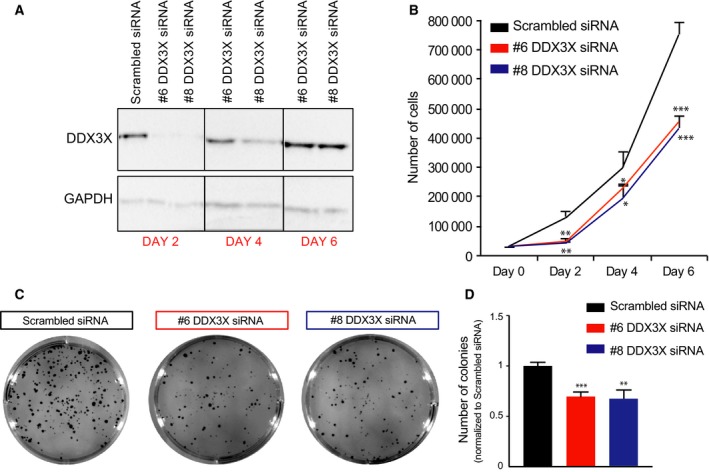
The DDX3X is required for breast cancer cells proliferation. (A) Western blot showing the protein levels of DDX3X and GAPDH in MCF7 cells transfected with either scrambled siRNA or one of two different siRNAs targeting DDX3X (#6 or #8). Cells were harvested at the indicated time points. Re‐expression of DDX3X at day 4 is presumably due to loss of the transfected siRNAs. (B) Proliferation curves for MCF7 cells treated as in A. Equal numbers of cells (3 × 10^4^) were seeded for proliferation assays 24 h after transfection (day 0) and then counted at the indicated time points. (C) Clonogenic assay of MCF7 cells treated as in A: 24 h after transfection, cells were seeded for clonogenic growth and stained 6 days after seeding. Images from a representative experiment are shown. (D) Quantification of number of colonies from C. *P*‐values represent statistical significance calculated with unpaired t test comparing to scrambled siRNA condition (*P*‐values: ns. >0.05; *≤0.05; **≤0.01; ***≤0.001).

MCF7 cells showed high sensitivity to DDX3X knockdown using two independent siRNAs (Fig. 1A,B). Seventy two hours after transfection with siRNA (i.e. day 2 after seeding – see Material and Methods), when depletion of DDX3X was virtually complete (Fig. 1A), DDX3X‐depleted MCF7 cells showed significantly impaired growth compared to control cells transfected with scrambled siRNA (Fig. 1B). In contrast, DDX3X depletion in nontumourigenic breast epithelial cells (MCF10A) showed little effect (Fig. S1).

We next performed a clonogenic assay in MCF7 cells upon knockdown of DDX3X using the same siRNAs. As shown, depletion of DDX3X significantly reduced the number of colonies formed (Fig. 1C,D).

### DDX3X regulates cell cycle in MCF7 cells

**Figure 2:**
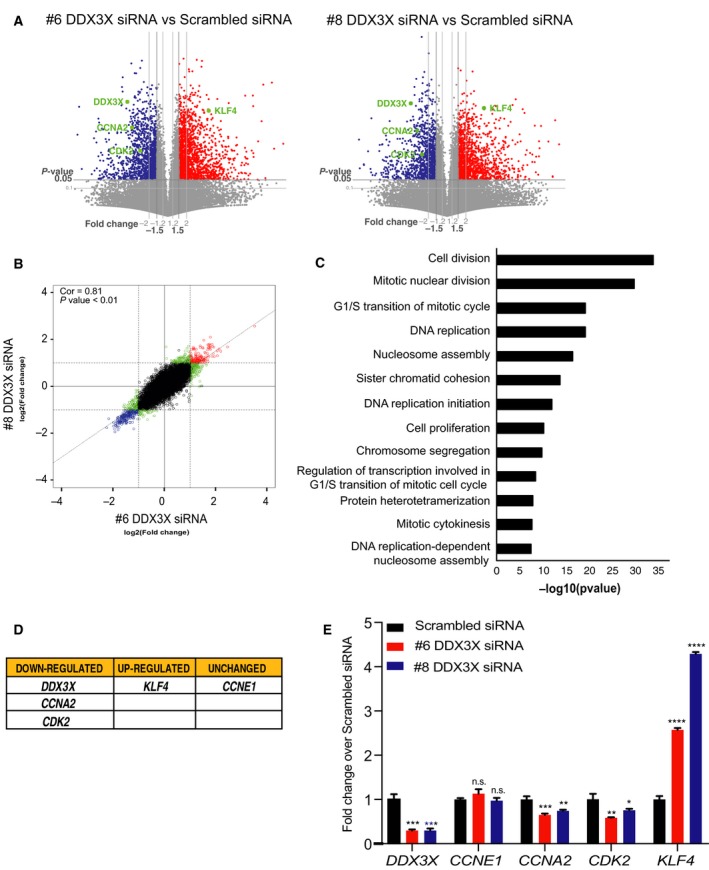
Gene expression changes upon knockdown of DDX3X in MCF7 cells. (A) Volcano plots for each individual differential expression analysis showing gene expression changes in MCF7 cells depleted of DDX3X with either DDX3X targeting siRNAs #6 or DDX3X targeting siRNAs #8. Significant genes (based on the specified threshold values) are indicated by red (up‐regulated) and blue (down‐regulated) points. Genes that are not changing in expression are coloured grey. (B) Correlation plot between the log‐fold change for comparison of the two siRNAs used to knockdown DDX3X (#6 and #8). (Red = genes up‐regulated in both conditions; Blue = genes down‐regulated in both conditions; Green = genes significant in one condition or the other; Black = not significant genes.) The corresponding Pearson correlation (‘Cor’) and *P*‐value for a linear model (‘*P* val’) are shown. (C) GO term analysis of down‐regulated genes upon knockdown of DDX3X in MCF7 cells. Down‐regulated genes in knockdown DDX3X MCF7 cells were identified from RNA‐seq data as described in Materials and methods. The GO enrichment of down‐regulated genes was obtained using the DAVID functional annotation tool with default settings. The *P*‐values were calculated using the ‘default’ method of DAVID (*P*‐Value ≤0.01). *Y*‐axis shows the GO terms related to biological processes in DDX3X knockdown MCF7 cells; X‐axis shows –log_10_ *P*‐values of each GO term. (D‐E) Validation of RNA‐seq upon DDX3X knockdown in MCF7 cells. (D) Table showing selected genes for RT‐qPCR validation: those transcripts resulted down‐regulated (*DDX3X, CCNA2, CDK2*), up‐regulated (*KLF4*) or unchanged (*CCNE1*) in RNA‐seq analysis of DDX3X knockdown MCF7 cells. (E) Levels of transcripts (listed in Table, panel D) in MCF7 cells transfected with either scrambled siRNA or one of two different siRNAs targeting DDX3X (#6 or #8), measured by RT‐qPCR. Cells were harvested 72 h after transfection. Results represent the average of three replicates. *P*‐values represent statistical significance calculated with unpaired t test comparing to scrambled siRNA condition (*P*‐values: ns. > 0.05; *≤0.05; **≤0.01; ***≤0.001; ****≤0.0001).

The importance of DDX3X in the proliferation of MCF7 breast cancer cells suggested a relevant and specific biological role for DDX3X in the growth of this cell line. To examine which pathways might be linked to reduced proliferation observed upon depletion of DDX3X, we performed RNA‐seq analysis of MCF7 cells following knockdown of DDX3X with each siRNA (#6 and #8) and compared the results to those obtained from RNA‐seq of control scrambled siRNA cells. These analyses revealed that knockdown of DDX3X caused significant gene expression changes (Fig. 2A; Supporting Information SF2) with good reproducibility between the two knockdown siRNAs used (Pearson = 0.81) (Fig. 2B). A gene ontology search using DAVID functional annotation tool was used to categorize the differentially expressed genes. Figure 2C shows the GO terms related to biological processes for genes down‐regulated upon DDX3X knockdown in MCF7 cells. This analysis indicated that genes encoding proteins involved in cell cycle were down‐regulated upon knockdown of DDX3X in MCF7 cells.

We next validated, by qPCR, a subset of cell cycle relevant genes whose expression was altered upon DDX3X knockdown in MCF7 cells. As shown in Fig. 2D,E, expression of these genes was altered exactly as predicted from the RNA‐seq data: *CCNA2* and *CDK2* expression was reduced upon DDX3X knockdown, whilst *CCNE1* expression was unchanged and *KLF4* expression was increased.

**Figure 3:**
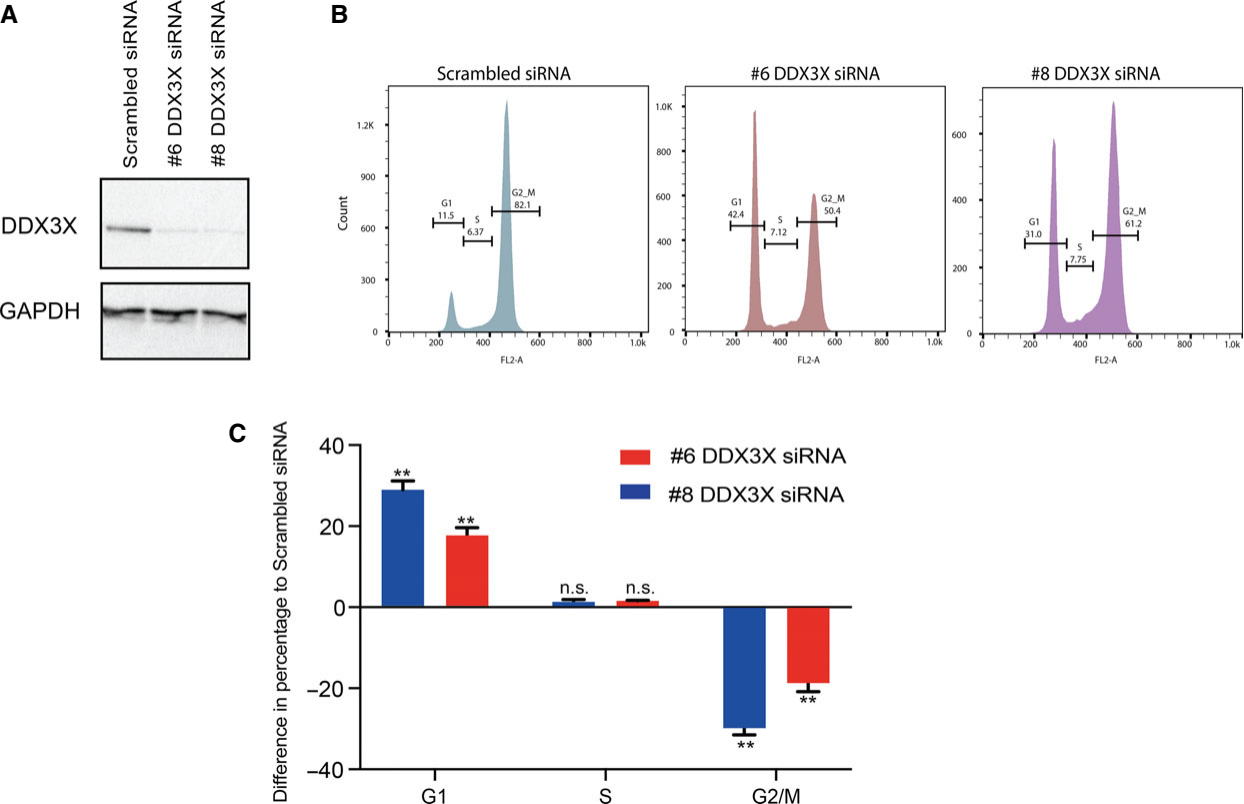
DDX3X knockdown arrests MCF7 cells in G1 phase. MCF7 cell cycle progression flow cytometric analysis based on PI staining in MCF7 cells 72 h after transfection with either scrambled siRNA or one of two different siRNAs targeting DDX3X (#6 or #8). (A) Western blots showing protein levels of DDX3X and GAPDH in MCF7 cells transfected with scrambled siRNA or one of two different siRNAs targeting DDX3X (#6 or #8). (B) Representative images from FlowJo analysis of flow cytometric analysis of cell cycle progression upon PI staining (quantified in C). (C) Quantification of the data in B for each phase (G1‐S‐G2M) plotted as the difference in percentages for each DDX3X siRNA used to those of the scrambled siRNA values. Results represent the average of two replicates. *P*‐values represent statistical significance calculated with unpaired *t* test comparing to scrambled siRNA condition (*P*‐values: ns. >0.05; **≤0.01).

Given that the results from the RNA‐seq analysis highlighted altered expression of cell cycle related genes in DDX3X‐deficient cells, we next analysed cell cycle progression of MCF7 cells upon DDX3X knockdown. In agreement with our RNA‐seq analysis, knockdown of DDX3X in MCF7 cells caused a dramatic accumulation of cells in G1 phase, indicating an impairment of cell cycle progression to DNA replication (Fig. 3A–C).

Taken together, these data indicate that DDX3X exerts regulatory activity on the cell cycle of MCF7 cells, and that its loss impairs progression of cells into S phase.

### KLF4 mediates DDX3X‐dependent regulation of MCF7 cell cycle

Transcriptome and cell cycle analysis upon depletion of DDX3X highlighted the role of this helicase in cellular proliferation and cell cycle progression in MCF7 breast cancer cells. Interestingly, whilst cell cycle effector genes, such as cyclins and cyclin‐dependent kinases, were down‐regulated upon knockdown of DDX3X, expression of the negative regulator of the cell cycle, *KLF4* 35, was dramatically increased in MCF7 cells (Fig. 2E) as well as in more aggressive MDA‐MB‐231 breast cancer cells (Fig. S3). We therefore considered whether increased expression of KLF4 could mediate the growth and cell cycle effects observed upon knockdown of DDX3X.

**Figure 4:**
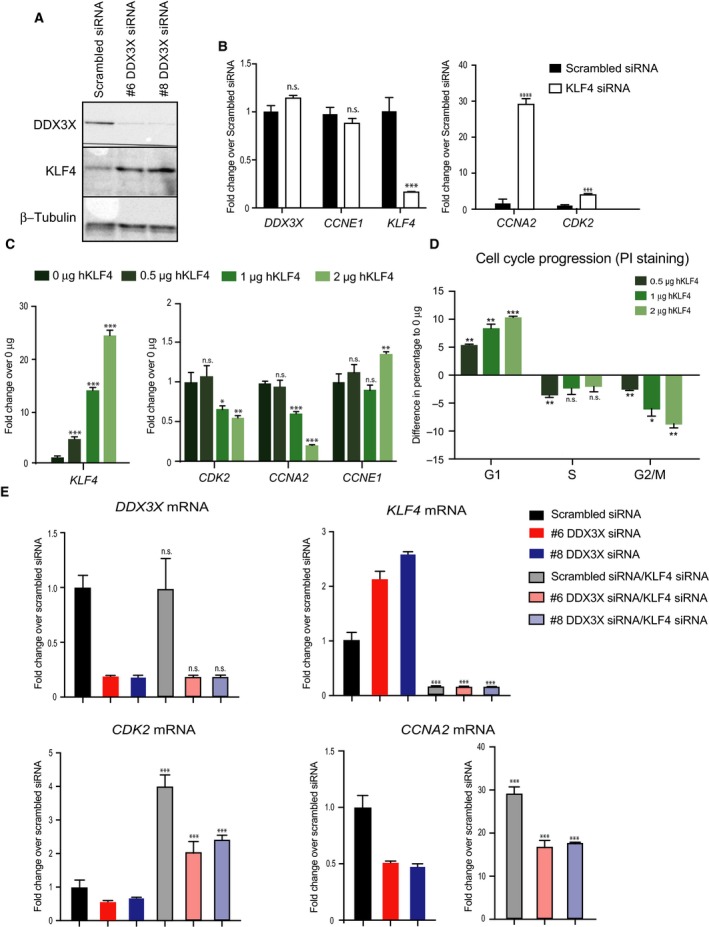
Kruppel‐like factor 4 mediates DDX3X‐dependent regulation of cell cycle. (A) Western blot showing the protein levels of DDX3X, KLF4 and β‐tubulin in MCF7 cells transfected with either scrambled siRNA or one of two different siRNAs targeting DDX3X (#6 or #8). (B) mRNA levels of *DDX3X, CCNE1, KLF4, CCNA2* and *CDK2* in MCF7 cells transfected with either scrambled siRNA or KLF4 targeting siRNA, measured by qPCR. Cells were harvested 72 h after transfection. Results represent the average of three replicates. (C) mRNA levels of *DDX3X*,*CCNE1, KLF4, CCNA2* and *CDK2* in MCF7 cells transfected with increasing amounts of a *hKLF4* expressing plasmid (as described in Materials and methods), measured by RT‐qPCR. Cells were harvested 48 h after transfection. Results represent the average of three replicates. (D) Cell cycle progression flow cytometric analysis in MCF7 cells transfected with increasing amount of a *hKLF4* expressing plasmid (as in C). Shown is the difference in percentages for each phase (G1–S–G2M) plotted as the difference in percentages for each condition (0.5 μg hKLF4, 1 μg hKLF4, 2 μg hKLF4) to those of the 0 μg hKLF4 control values. Cells were harvested 48 h after transfection. Results represent the average of two replicates. *P*‐values represent statistical significance calculated with unpaired *t* test comparing to scrambled siRNA or 0 μg conditions (*P*‐values: ns. >0.05; **≤0.01; ***≤0.001). (E) Double knockdown of *DDX3X* and *KLF4* in MCF7 cells: levels of *DDX3X*,*KLF4, CDK2* and *CCNA2* transcripts in MCF7 cells transfected with either scrambled siRNA or #6 *DDX3X* siRNA or #8 *DDX3X* siRNA alone or combined with *KLF4* targeting siRNA, measured by RT‐qPCR. Cells were harvested 72 h after transfection. Results represent the average of two replicates. *P*‐values represent statistical significance calculated with unpaired t test comparing the double transfection conditions to the single transfection respective condition (*P*‐values: ns. >0.05; *≤0.05; **≤0.01; ***≤0.001; ****≤0.0001).

We confirmed that DDX3X knockdown enhances expression of *KLF4* not only at the mRNA level (Fig. 2E) but also at the protein level in MCF7 cells (Fig. 4A). In fact, the KLF4 transcription factor was relatively weakly expressed in control MCF7 cells (transfected with scrambled siRNA), but it was robustly induced upon DDX3X knockdown. In line with these results, we reasoned that DDX3X modulates the cell cycle of MCF7 cells, at least in part, by maintaining relatively low levels of KLF4 which in turn allows expression of cell cycle effector proteins to promote cell cycle progression. To test this hypothesis, we decided to focus on the link between KLF4 up‐regulation and the down‐regulation of cyclins and cyclin‐dependent kinases detected upon DDX3X knockdown, which would ultimately result in the observed G1 arrest (Figs 2E and 3).

If the phenotype observed upon DDX3X knockdown is due to up‐regulation of KLF4, then depletion of KLF4 should increase expression of *CCNA2* and *CDK2*. Conversely, overexpression of *KLF4* should phenocopy the effects caused by knocking down DDX3X. As shown in Fig. 4B, knockdown of KLF4 in MCF7 cells enhanced expression of *CCNA2* and *CDK2*. In contrast, *CCNE1* gene expression, which was unchanged when DDX3X was depleted, also remained unchanged upon KLF4 knockdown. Importantly, *DDX3X* levels did not change upon KLF4 knockdown. Moreover, *KLF4* overexpression affected the expression of these genes in a similar manner to that observed upon DDX3X knockdown (Figs 2E and 4C). Indeed, the expression of genes that were down‐regulated upon DDX3X knockdown (*CCNA2, CDK2*) was decreased proportionally to the level of *KLF4* overexpression. *CCNE1* gene expression, which was unchanged in DDX3X knockdown, also showed no change upon *KLF4* overexpression (Fig. 4C). These results are entirely consistent with the notion that the effects observed upon DDX3X knockdown are largely due to elevated expression of KLF4 in MCF7 breast cancer cells.

We then investigated whether overexpression of KLF4 would phenocopy the effect of DDX3X depletion on cell cycle progression of MCF7 cells. As expected, overexpression of KLF4 caused accumulation of cells arrested in G1 phase (Fig. 4D), similar to that observed upon DDX3X knockdown (Fig. 3). Finally, we examined whether depletion of both DDX3X and KLF4 would rescue the down‐regulation of *CDK2* and *CCNA2* cell cycle genes, observed upon knockdown of DDX3X alone. Importantly, depletion of both DDX3X and KLF4 in MCF7 cells partially restored the expression of both *CDK2* and *CCNA2* genes (Fig. 4E).

Overall, these findings show that up‐regulation of KLF4, induced by depletion of DDX3X expression in MCF7 cells, very likely drives the cell cycle arrest observed upon DDX3X knockdown. Furthermore, our data indicate that DDX3X's cell cycle regulatory activity may function directly through the DDX3X‐dependent inhibition of KLF4 transcription factor expression.

**Figure 5:**
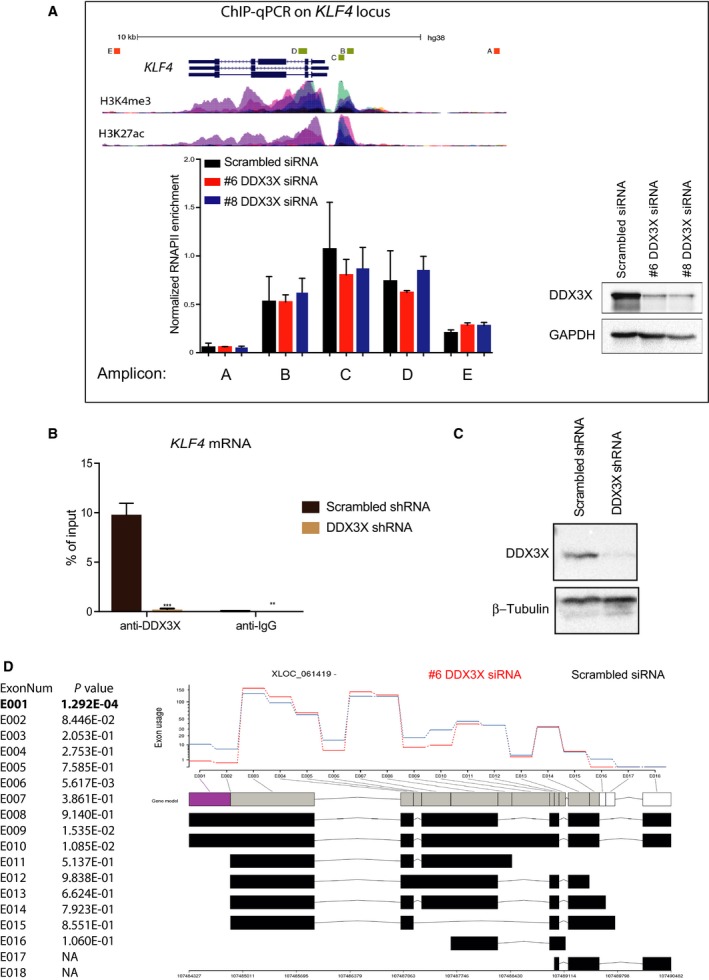
DDX3X binds *KLF4* mRNA and regulates its splicing. (A) Chromatin immunoprecipitation (ChIP)–qPCR analysis of RNA polymerase II (RNAPII) at the *KLF4* gene in control (black bars) and DDX3X knockdown (red and blue bars) cells. ‘Normalized RNAPII enrichment’ ChIP efficiency calculated as a percentage of input normalized to the internal control for RNAPII occupancy, represented in this case by a *GAPDH* house‐keeping gene promoter region. A schematic representation of the *KLF4* gene is shown (note: it is transcribed from right to left), and H3K4me3 and H3K27ac levels (from ENCODE) are included for reference purposes. The position of the amplicons analysed (A–E) are indicated. A western blot showing the levels of DDX3X in knockdown cells (#6 and #8 DDX3X siRNAs) is shown to the right. (B) CLIP‐qPCR was performed as described in Materials and methods with anti‐DDX3X and anti‐IgG antibodies in MCF7 cells transduced with either scrambled shRNA or DDX3X shRNA. RNA obtained from each immunoprecipitation was analysed by RT‐qPCR to determine enrichment of *KLF4* mRNA. Results represent the average of three replicates. *P*‐values represent statistical significance calculated with unpaired t test comparing to scrambled shRNA condition (*P*‐values: ns. > 0.05; *≤0.05; **≤0.01; ***≤0.001). (C) Western blot showing the protein levels of DDX3X and β‐tubulin in MCF7 cells used in B. (D) Differential exon usage analysis in *KLF4* mRNA upon *DDX3X* knockdown. Gene model colour code from purple to white indicates the descending statistical significance of the difference observed for each exon usage (purple: *P*‐value ≤5.0E‐4; grey box: *P*‐value >5.0E‐4; white box: N.A.). Analysis was obtained using DEXseq (Cut‐off *P*‐value ≤5.0E‐4); Top panel = normalized counts average of replicates for each sample; bottom panel = flattened gene model highlighting alternative annotated transcripts. (Red) Data from #6 DDX3X siRNA knockdown samples; (Blue) scrambled control. For each significant gene, all of the exons across all isoforms are labelled (E001, E002, etc.) and the ‘exon usage’ (essentially a normalized read count at that exon) is plotted for each of the conditions (averaged between the replicates). An exon (E001) that shows a significant difference between the conditions is highlighted in purple (*P*‐value ≤5.0E‐4).

Since the level of KLF4 mRNA and protein are affected by DDX3X expression (Figs 2E and 4A), we first tested whether knockdown of DDX3X induces *KLF4* expression at the level of transcription. To do so, we used an anti‐RNA polymerase II antibody in chromatin immunoprecipitation experiments and examined polymerase occupancy at the *KLF4* gene in control and DDX3X knockdown cells (Fig. 5A). We observed no effect of depleting DDX3X at any of the amplicons analysed (Fig. 5A). These results indicate that DDX3X depletion does not induce expression of KLF4 by increasing its rate of transcription.

Next, we reasoned that the helicase could regulate KLF4 function post‐transcriptionally by directly binding to its mRNA. We therefore performed UV cross‐linking immunoprecipitation (CLIP) to test for direct interaction between DDX3X protein and *KLF4* mRNA. As shown (Fig. 5B,C), *KLF4* mRNA was specifically enriched in DDX3X immunoprecipitates in MCF7 cells expressing normal levels of DDX3X. Importantly, binding of DDX3X to *KLF4* mRNA was lost when DDX3X was knocked‐down (Fig. 5B,C), testifying to the specificity of the assay. Moreover, specificity was further confirmed by showing that another helicase protein, DDX24, does not bind to *KLF4* mRNA, whilst neither DDX3X nor DDX24 bound to a control mRNA, *PUS1* mRNA (Fig. S4). Thus, DDX3X directly interacts with *KLF4* transcripts.

Given this, we next tested the hypothesis that DDX3X regulates *KLF4* mRNA splicing by analysing the effect of DDX3X depletion upon exon usage in *KLF4* mRNA. This identified significant differences in the usage of a number of exons, particularly in the inclusion of exon 1 (Fig. 5D). These observations suggest that DDX3X regulates the selection of certain exons during *KLF4* mRNA splicing, thereby regulating production of specific *KLF4* isoforms.

Taken together, our findings reveal a robust negative effect of DDX3X on the expression of *KLF4* mRNA and protein and they suggest that DDX3X's biological function in MCF7 breast cancer cells is linked to its direct binding to *KLF4* transcripts.

## Discussion

In the present work, we show that DDX3X is required for efficient proliferation of MCF7 breast cancer cells and for their progression through the cell cycle, specifically into S phase. This confirms similar findings from other groups 26, 27. Moreover, a similar role for DDX3X in regulating the cell cycle was previously reported in lung cancer cells and colorectal cancer cells in which depletion of DDX3X significantly reduced proliferation and caused a G1 arrest 22, 23. In a separate study, DDX3X regulation of *CCNE1* mRNA translation was responsible for progression of HeLa cells to S phase 36. In this cell line, DDX3X was required for production of CCNE1 protein but not for *CCNE1* mRNA levels. Consistent with this, our data show that the mRNA level of *CCNE1* is not affected by DDX3X knockdown in MCF7 cells. Instead, expression of positive cell cycle regulators *CCNA2* and *CDK2* was reduced and expression of a negative master regulator of the cell cycle, *KLF4*, was significantly increased in DDX3X‐depleted MCF7 cells. Moreover, we show that DDX3X directly interacts with *KLF4* mRNA and that KLF4 protein levels are strongly increased upon DDX3X knockdown. Indeed, we provide evidence that DDX3X regulates *KLF4* mRNA alternative splicing in a way that would likely affect its protein production. Although our ChIP experiments indicate that DDX3X depletion does not affect *KLF4* gene transcription, it remains a possibility that DDX3X regulates *KLF4* splicing in a cotranscriptional manner.

**Figure 6:**
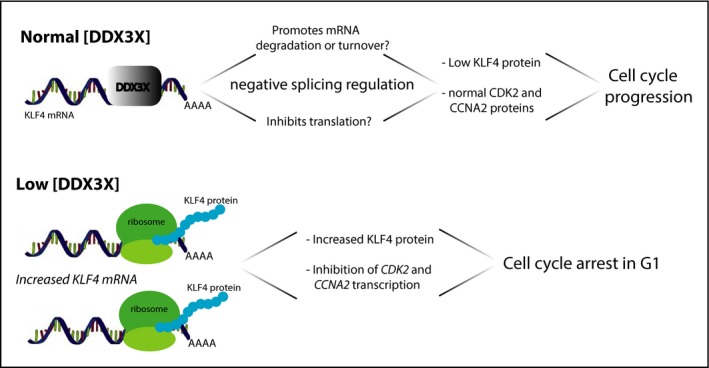
Model of DDX3X‐dependent regulation of cell cycle through repression of KLF4 expression.

We propose a model in which DDX3X exerts oncogenic activity by binding to *KLF4* mRNA to inhibit its correct splicing thereby promoting expression of key cell cycle genes (e.g. *CCNA2* and *CDK2*) (Fig. 6). Based on our observations, we speculate that binding of DDX3X, which leads to an altered splicing pattern of *KLF4* mRNA, produces an unstable RNA isoform that is then targeted for degradation. Alternatively, but not necessarily mutually exclusively, DDX3X may directly inhibit translation of *KLF4* mRNA. This would be consistent with the reported ability of DDX3X to interact with members of the translational machinery 37, 38, 39, 40.

In our model, we propose that the cell cycle regulatory activity of DDX3X is exerted through its regulation of *KLF4* mRNA and protein levels. In agreement with this, we observed that knockdown of KLF4 induced expression of *CCNA2* and *CDK2* genes that were down‐regulated by DDX3X knockdown. When *KLF4* was overexpressed in MCF7 cells, expression of *CCNA2* and *CDK2* was reduced proportionally to the level of expression of *KLF4* and consistent with the observed increased cell cycle arrest in G1. Moreover, when both DDX3X and KLF4 were depleted, the levels of *CDK2* and *CCNA2* expression were partially restored compared to the down‐regulation observed upon knockdown of DDX3X alone. However, the higher *CDK2* and *CCNA2* expression levels detected with *KLF4* siRNA alone compared with *DDX3X* and *KLF4* siRNAs together, suggests that DDX3X might also regulate expression of *CDK2* and *CCNA2* mRNAs through additional mechanisms, independently of KLF4.

Association between Cyclin A2 (encoded by *CCNA2*) and CDK2 is necessary for cell cycle progression into S phase and it is specifically required for DNA replication 41, 42, 43, 44. Interestingly, among the genes down‐regulated upon DDX3X knockdown in MCF7 cells, many genes encode replication‐related factors such as the replication origin recognition complex 1 (ORC1), factors involved in DNA replication regulation (CDC6 and CDC45) and chromosome‐related factors such as minichromosome maintenance proteins (MCM2, 3, 5, 7, 8,10) that associate with chromatin in G1 (Fig. S2). This suggests DDX3X may have a broad role in regulating S phase‐relevant factors.

DEAD box proteins have been suggested to exert both oncogenic and tumour‐suppressive roles, in a context‐ and cancer‐dependent fashion and through different mechanisms that affect a wide range of molecular events, from transcription to translation 13, 19. These observations have highlighted DDX enzymes as potential candidates for therapeutic development as well as disease biomarkers 13. Inhibitors of NTPase activity (like nucleoside analogues), which affect RNA binding and helicase activity of DEAD‐box proteins, have already been designed, tested and shown to possess anticancer potential 45. Our findings that up‐regulation of *KLF4* mRNA upon DDX3X knockdown in both MCF7 cells and MDA‐MB 231 cells suggests that the DDX3X:KLF4 axis of cell cycle regulation could be a common feature of breast cancer cell lines. Furthermore, the fact that DDX3X possesses a proproliferative function in breast cancer cells but not in nontumourigenic breast epithelial cells 26, highlights this RNA helicase as a potential therapeutic target in the fight against breast cancer.

## Author contributions

EC and AJB designed experiments and interpreted results. EC and AA carried out experiments, and NH performed bioinformatical analysis of data. TK and AJB supervised the project. EC and AJB wrote the manuscript with contributions from all authors.

## Supporting information


**Fig. S1.** MCF10A proliferation is not affected by DDX3X knock‐down.

**Fig. S2.** DDX3X knockdown differential gene expression analysis.

**Fig. S3.** DDX3X knockdown causes up‐regulation of *KLF4* in MDA‐MB‐231 breast cancer cells.

**Fig. S4.** CLIP–qPCR in wild‐type MCF7 cells.
