# Supplementary material for: DDX3X RNA helicase affects breast cancer cell cycle progression by regulating expression of KLF4
Source: FEBS Lett. 2018 Jun 21;592(13):2308–22. doi: 10.1002/1873-3468.13106 (PMC6100109; doi:10.1002/1873-3468.13106)
Supplement: Supplementary file 1 — Fig. S1. MCF10A proliferation is not affected by DDX3X knock‐down. [file FEB2-592-2308-s001.pdf]

Supporting Information SF1

A

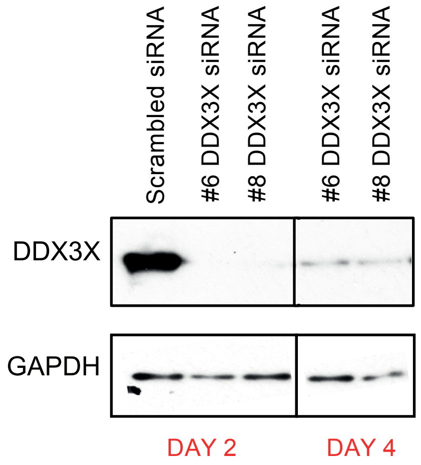

B

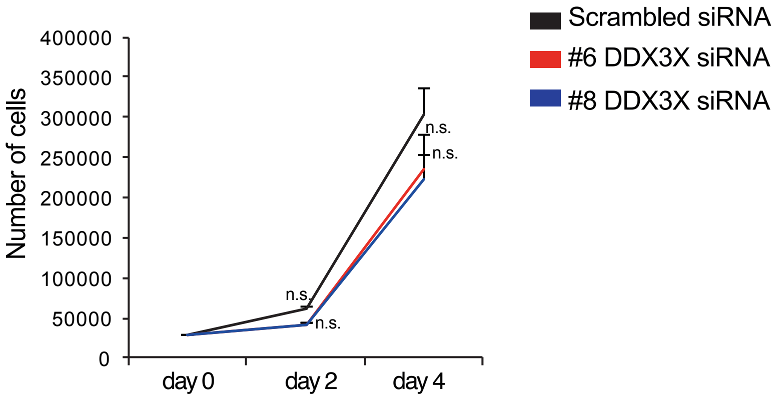

SF1. MCF10A proliferation is not affected by DDX3X knock-down. (A) Western blot showing the protein levels of DDX3X and GAPDH in MCF10A cells transfected with either scrambled siRNA or one of two different siRNAs targeting DDX3X (#6 or #8) and harvested at the indicated time-points. Partial recovery of DDX3X expression is evident at day 4. This is presumably due to loss of the transfected siRNAs after this time. (B) Proliferation curves for MCF10A cells treated as in A. Cells were seeded for proliferation assays 24h after transfection (day 0) and counted at the indicated time-points. P-values represent statistical significance calculated with unpaired t test compared to scramble siRNA (P-values: ns. > 0.05; * ≤ 0.05; ** ≤ 0.01; *** ≤ 0.001; **** ≤ 0.0001)
