# Supplementary material for: DDX3X RNA helicase affects breast cancer cell cycle progression by regulating expression of KLF4
Source: FEBS Lett. 2018 Jun 21;592(13):2308–22. doi: 10.1002/1873-3468.13106 (PMC6100109; doi:10.1002/1873-3468.13106)
Supplement: Supplementary file 2 — Fig. S2. DDX3X knockdown differential gene expression analysis. [file FEB2-592-2308-s002.pdf]

Supporting Information SF2

Supplementary Material - DDX3X knockdown Differential Gene Expression Analysis

Differential Gene Expression Analysis of DDX3X knockdown as separate analysis of #6 DDX3X siRNA vs Scrambled siRNA and #8 DDX3X siRNA vs Scrambled siRNA, is available at the following link:

https://figshare.com/articles/DDX3X_RNA-seq_-_Differential_Gene_Expression_Analysis/6230816
