# Supplementary material for: DDX3X RNA helicase affects breast cancer cell cycle progression by regulating expression of KLF4
Source: FEBS Lett. 2018 Jun 21;592(13):2308–22. doi: 10.1002/1873-3468.13106 (PMC6100109; doi:10.1002/1873-3468.13106)
Supplement: Supplementary file 3 — Fig. S3. DDX3X knockdown causes up‐regulation of KLF4 in MDA‐MB‐231 breast cancer cells. [file FEB2-592-2308-s003.pdf]

Supporting Information SF3

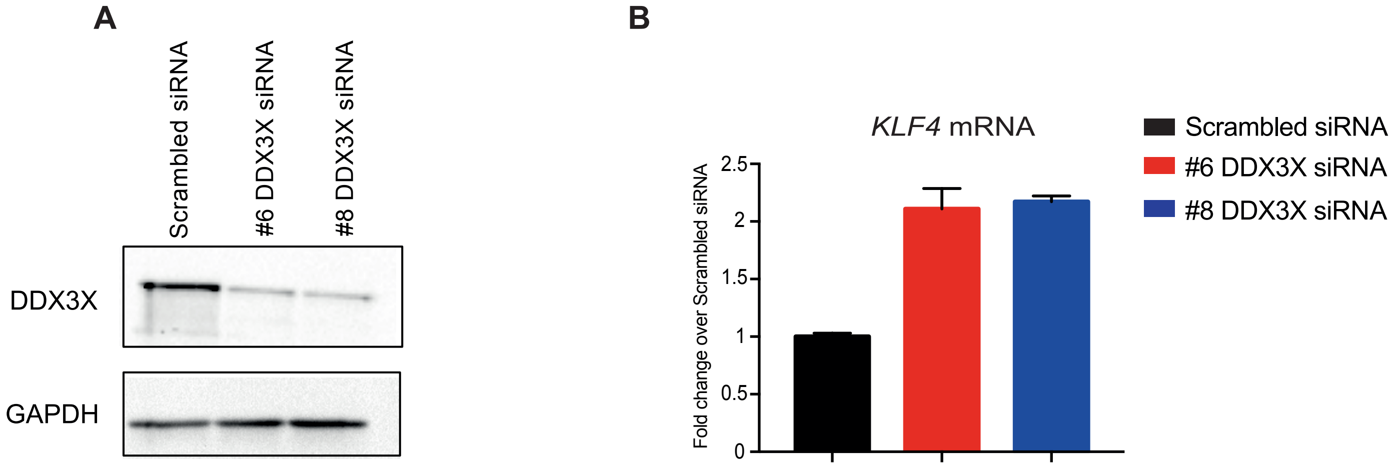

SF3. DDX3X knockdown causes up-regulation of *KLF4* in MDA-MB-231 breast cancer cells. (A) Western blot showing the protein levels of DDX3X and GAPDH in MDA-MB-231 cells transfected with either scrambled siRNA or one of two different siRNAs targeting DDX3X (#6 or #8). (B) Level of *KLF4* transcript in MDA-MB-231cells treated as in A, measured by RT-qPCR. Cells were harvested 72h after transfection. Results represent the average of two replicates.
