# Supplementary material for: DDX3X RNA helicase affects breast cancer cell cycle progression by regulating expression of KLF4
Source: FEBS Lett. 2018 Jun 21;592(13):2308–22. doi: 10.1002/1873-3468.13106 (PMC6100109; doi:10.1002/1873-3468.13106)
Supplement: Supplementary file 4 — Fig. S4. CLIP–qPCR in wild‐type MCF7 cells. [file FEB2-592-2308-s004.pdf]

Supporting Information SF4

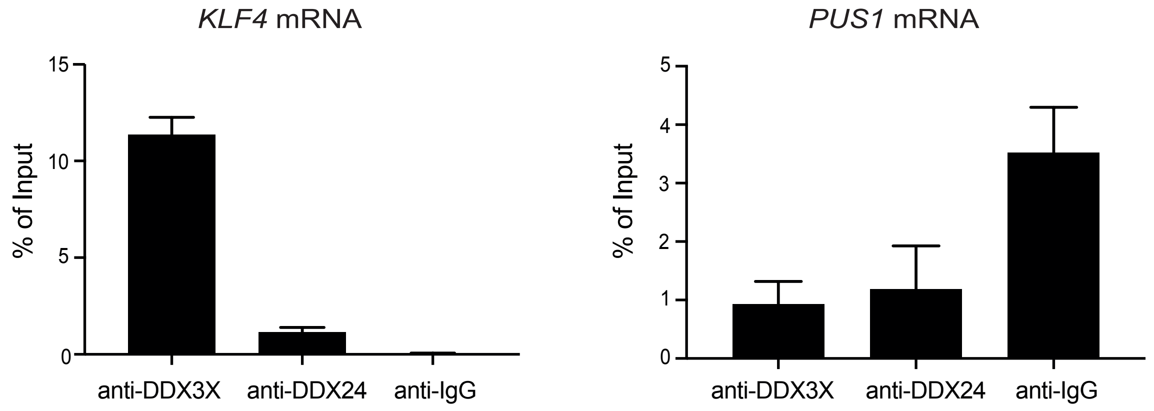

SF4. CLIP-qPCR in wild type MCF7 cells. CLIP-qPCR was performed as described in Materials and Methods with anti-DDX3X, anti-DDX24 and anti-IgG antibodies in wild type MCF7 cells. RNA obtained from each immunoprecipitation was analysed by RT-qPCR to determine enrichment of *KLF4* or *PUS1* mRNAs. Results represent the average of three replicates.
